# A case of hepatic anisakiasis caused by *Pseudoterranova decipiens* mimicking metastatic liver cancer

**DOI:** 10.1186/s12879-018-3540-8

**Published:** 2018-12-04

**Authors:** Yasuhiro Murata, Katsuhiko Ando, Masanobu Usui, Hiromu Sugiyama, Akinobu Hayashi, Akihiro Tanemura, Hiroyuki Kato, Naohisa Kuriyama, Masashi Kishiwada, Shugo Mizuno, Hiroyuki Sakurai, Shuji Isaji

**Affiliations:** 1 Department of Hepatobiliary Pancreatic and Transplant surgery, Mie University Graduate School of Medicine, 2-174 Edobashi, Tsu, Mie 514-8507 Japan; 2 Department of Medical Zoology, Mie University Graduate School of Medicine, Tsu, Japan; 3 Department of Parasitology, National Institute of Infectious Diseases, Tokyo, Japan; 4 Department of Pathology, Mie University Graduate School of Medicine, Tsu, Japan

**Keywords:** *Anisakis*, Extragastrointestinal anisakiasis, Hepatic anisakiasis, *Pseudoterranova decipiens*, Genetic examination, Metastatic liver cancer

## Abstract

**Background:**

Anisakid nematodes (*Anisakis* spp. or *Pseudoterranova* spp.) usually infect gastric or intestinal walls, while they rarely infect in extra-gastrointestinal sites of human body. Generally, Anisakis spp. larvae are highly infected in fish intermediate hosts, whereas Pseudoterranova spp. larvae are very rarely infected. To the best of our knowledge, there have been no reports which have documented cases of hepatic anisakiasis caused by *Pseudoterranova* spp. This report describes the first documented case of hepatic anisakiasis due to infection with *Pseudoterranova decipiens* and clinical features of the hepatic anisakiasis through literature review.

**Case presentation:**

The case was a 28-year-old man with prior history of malignancy who was found to have a hepatic mass mimicking metastatic liver tumor. A new low density area of 20 mm in diameter in liver segment 7 was found on follow-up CT. With suspicious diagnosis of metastatic liver cancer, laparoscopic partial hepatectomy was performed. A pathological examination revealed no evidence of malignancy, but showed necrotic granuloma with eosinophil infiltration and the presence of a larva with Y-shaped lateral cords, which are specific to anisakid larvae. The type of larva was identified as *Pseudoterranova decipiens* sensu *lato* using PCR of DNA purified from a fixed granuloma embedded in paraffin.

**Conclusion:**

The present report is the first to discuss the case of a patient with hepatic anisakiasis caused by *Pseudoterranova decipiens*. Hepatic anisakiasis is a potential differential diagnosis for hepatic tumors and genetic identification with the PCR method was reliable for obtaining final diagnosis even when the larvae body in the resected specimen collapses with time.

## Background

Anisakiasis is a foodborne disease caused by the accidental ingestion of larval nematodes belonging to the family Anisakidae [1, 2]. Incidence of anisakiasis, at one time relatively common only in East Asia due to the consumption of raw fish, have increased worldwide with the growing popularity of the seafood delicacy [3].

There are two forms, i.e., noninvasive and invasive, of anisakiasis. The noninvasive form is generally asymptomatic and involves no tissue penetration by the larvae. In case of invasive anisakiasis, anisakid larvae were usually found in the mucosa or submucosa of the gastric and intestinal walls, while they were less commonly detected in extra-gastrointestinal sites of human body [4]. Eosinophilic granuloma is commonly formed around embedded larva in the migrating sites and the histopathologic lesions are changed by the lapse of infection in the chronic invasive anisakiasis [5]. Incidental detection of this lesion is difficult to diagnose and differentiate from recurrence in patients with a prior history of malignancy. This may result in the need for resection, and in some cases the collapsing larvae body may make the definitive diagnosis difficult, even after resection.

Anisakid larvae is commonly classified into two types that have been implicated in human disease: *Anisakis* (sensu *lato*) and *Pseudoterranova* (sensu *lato*) [6]. Generally, Anisakis spp. larvae are highly infected in fish intermediate hosts, whereas Pseudoterranova spp. larvae are very rarely infected [7, 8]. *Anisakis* spp. are most often implicated in the invasive type of anisakiasis. In contrast, larvae belonging to the *Pseudoterranova* spp. are generally noninvasive, and extra-gastrointestinal anisakiasis due to *Pseudoterranova* spp. is extremely rare. In the present report, we discuss the case of a 28 year-old male with a prior history of malignancy who was diagnosed with an asymptomatic liver tumor mimicking metastatic liver cancer and received a final diagnosis of hepatic anisakiasis caused by *Psuedoterranova decipiens* through the process of genetic identification. The purpose of the present case study is to describe the first documneted case of hepatic anisakiasis due to infection with *Pseudoterranova decipiens* and assess clinical features of the hepatic anisakiasis through literature review.

## Case presentation

**Fig. 1:**
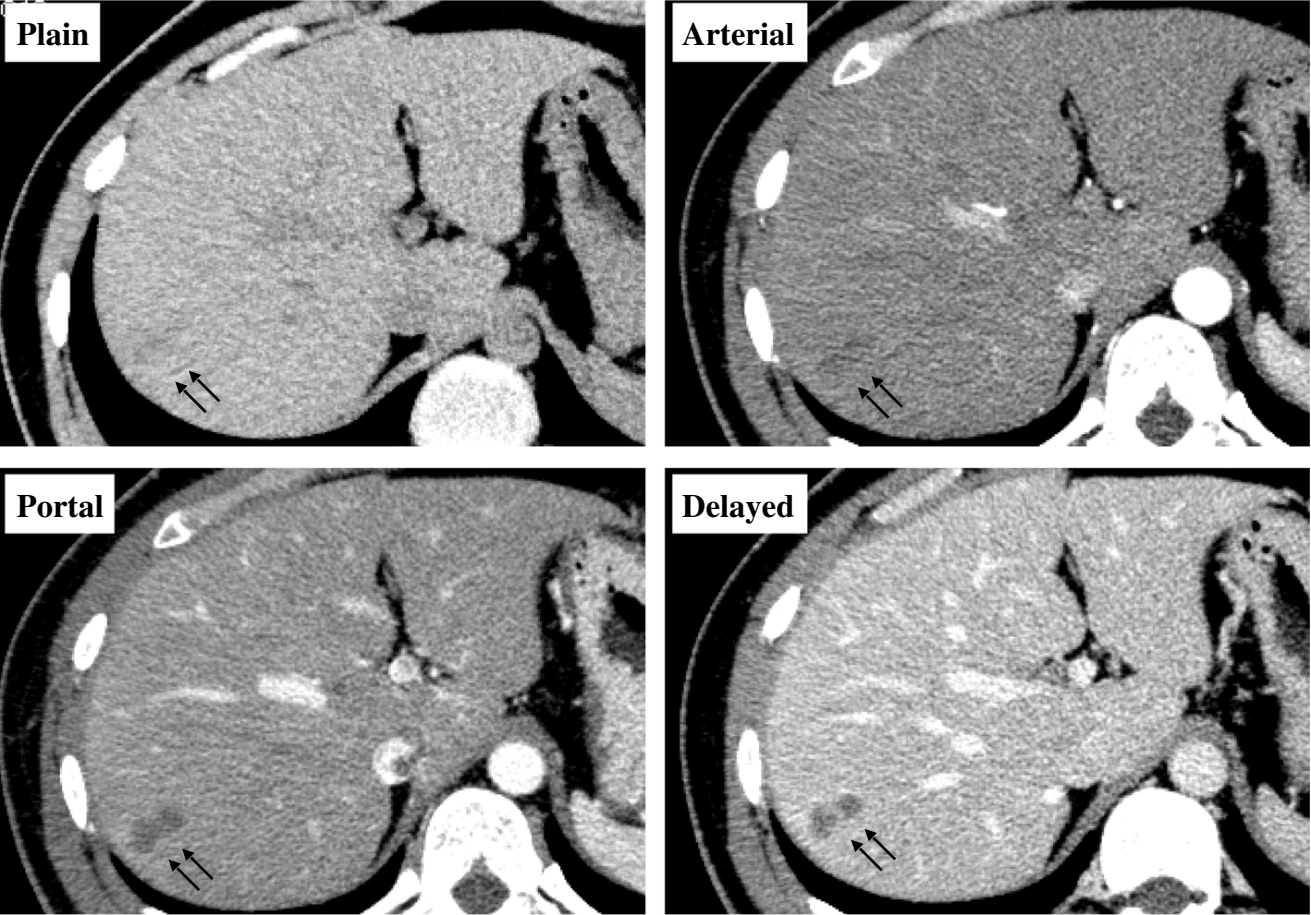
Axial slice on dynamic enhanced CT before operation. A solitary hypovascular mass detected in segment 7 of the liver (black arrows)

**Fig. 2:**
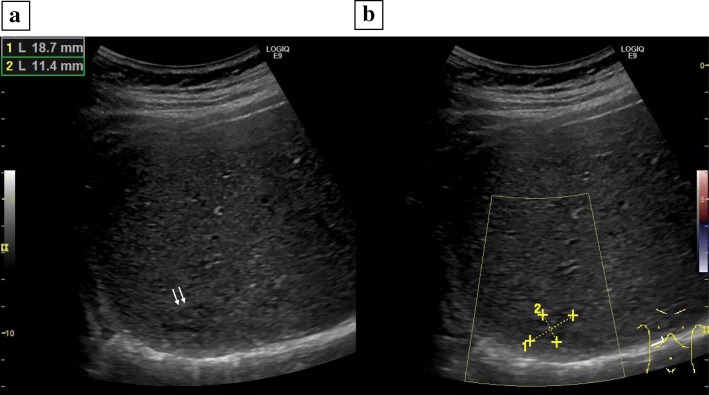
Findings of abdominal ultrasonography. **a** a 18.7 × 11.4 mm heterogeneous iso- and hypoechoic mass which displayed irregular shape and indistinct margin and included hyperechoic spots in segment 7 of the liver with the B-mode ultrasonography (white arrows), **b** The liver mass showed no flow signals in the color doppler mode

**Fig. 3:**
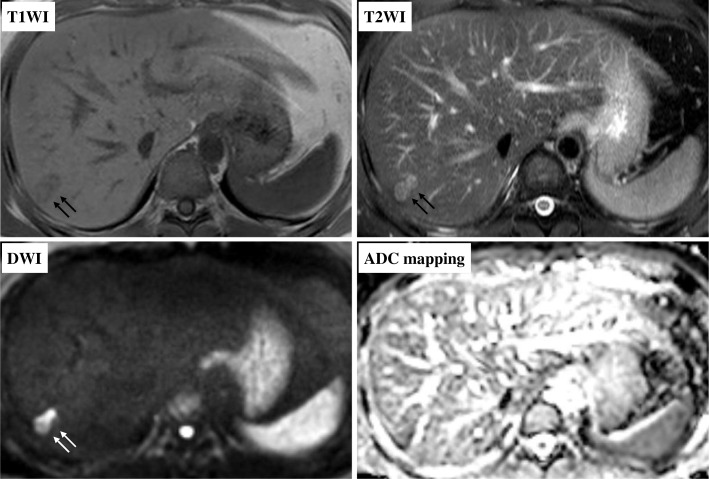
Axial slice on MRI before operation. The liver mass (dumbbell-shaped) had low signal intensity on T1-weighted images (T1WI), high signal intensity on T2-weighted images (T2WI), marked signal hyperintensity in diffusion-weighted imaging (DWI), and no signal hypointensity on ADC map

**Fig. 4:**
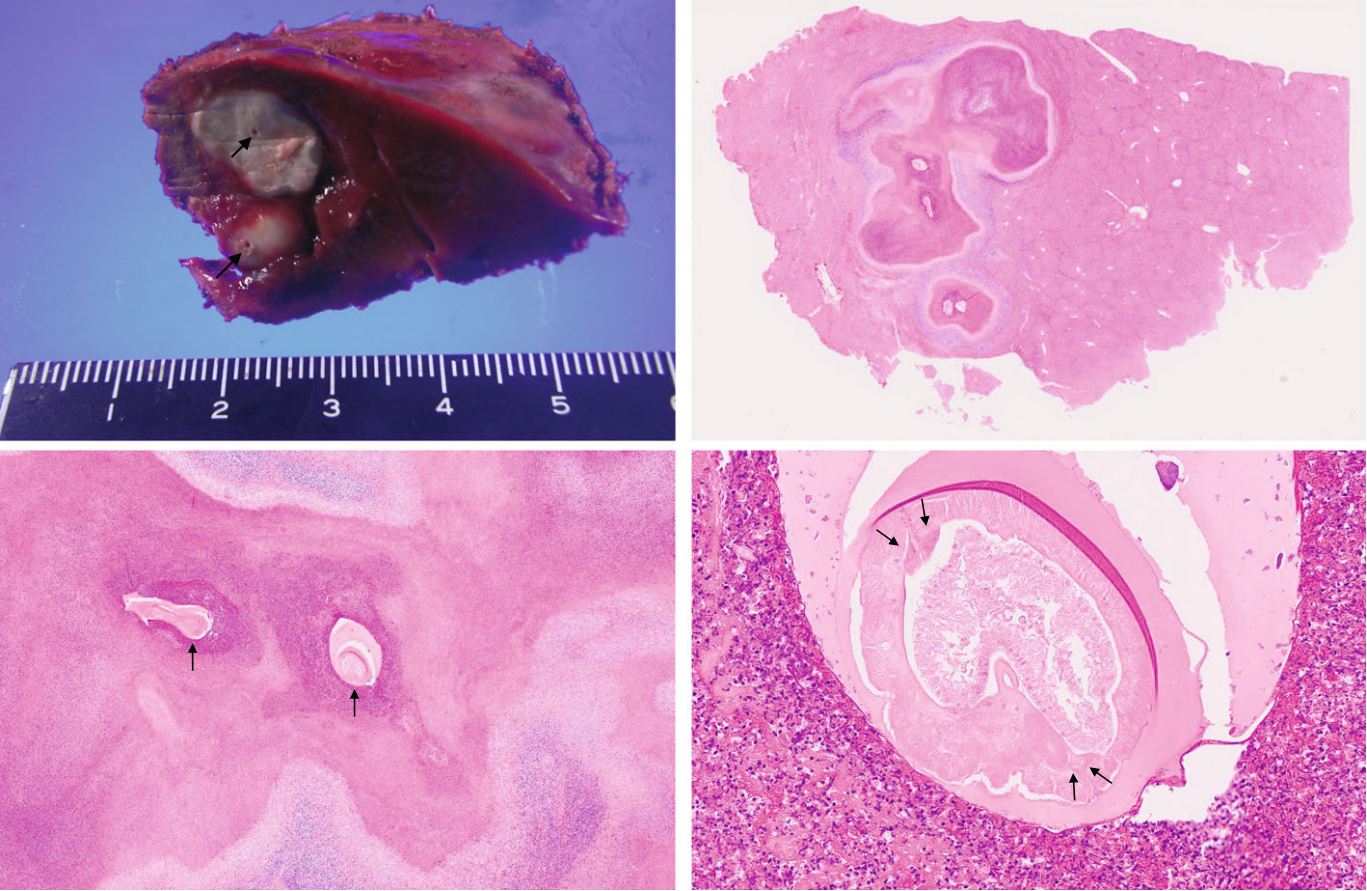
Macroscopic and microscopic findings of the resected specimen**.** The resected liver sample included a white nodule of 12 mm in diameter with a regular border macroscopically. A tiny pinhole was seen within the nodule (upper left, black arrow). Roupe finding showed epithelioid granuloma with central necrosis in the normal liver tissue (upper right, original magnification × 0.4). Within the central necrosis, a small hole which bears an exogeneous material was recognizable (lower left, brack arrow, original magnification × 2). Axial slice of the larva surrounded by epithelioid granuloma with infiltration of inflammatory cells such as monocytes and eosinophils. The larva body had collapsed, but the specific Y-shaped lateral cord was recognizable (lower right, black arrows, original magnification × 20)

**Fig. 5:**
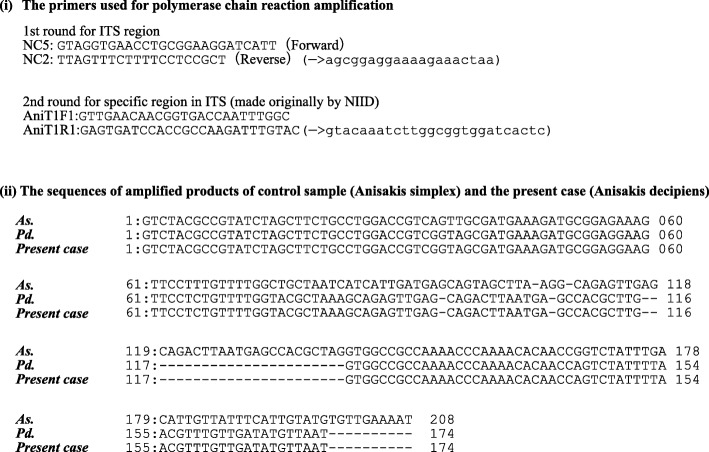
Genetic identification of anisakis and sequences obtained. As.: control sample of *Anisakis symplex*, Pd.: control sample of *Pseudoterranova decipiens* sensu *lato*

The patient is a 28 year-old male who had been diagnosed with right testicular cancer. He underwent right high orchidectomy in the department of nephro-urologic surgery in Mie University Hospital in Tsu city, Mie, Japan. The pathological diagnosis was seminoma, pT1N0M0, pStage IA according to the classification established by the Japanese Urological Association [9]. The risk status of tumor was classified as low risk by the International Germ Cell Consensus classification (IGCC) [10]. Thereafter, he received periodic check-ups. Follow-up CT performed at 3 months, 9 months, and 15 months after surgery did not indicate any abnormal findings suggesting recurrence and distant metastasis, but CT performed at 21 months after surgery detected a solitary dumbbell-shaped hypovascular tumor measuring 20 mm in Segment 7 of the liver (Fig. 1). Abdominal ultrasonography revealed an 18.7 × 11.4 mm heterogeneous iso- and hypoechoic mass which displayed an irregular shape and indistinct margin and included hyperechoic spots in segment 7 of the liver, and it did not show flow signal in color doppler mode (Fig. 2). Serum tumor markers, including CEA, CA19–9, AFP, PIVKA-II and hCG, were not found to be elevated. MRI showed a dumbbell-shaped liver tumor in segment 7 which had a low signal intensity on T1-weighted images (T1WI), high signal intensity on T2-weighted images (T2WI), marked signal hyperintensity in diffusion-weighted imaging (DWI), and no signal hypointensity on the ADC map (Fig. 3). PET-CT was performed to confirm the presence of a malignant liver tumor and to search for further metastases in the other organs, but the liver lesion had no specific 18fluoro-deoxyglucose (FDG) uptake compared with normal liver tissue. No other metastasis was detected. Metachronous liver metastasis of testicular cancer was suspected and laparoscopic partial hepatectomy of segment 7 was performed. The resected liver sample included a white nodule of 12 mm in diameter with a regular border in which a tiny pinhole was present macroscopically (Fig. 4). Microscopic examination showed epithelioid granuloma with central necrosis and infiltration of inflammatory cells such as monocytes and eosinophils infiltrated around the granuloma. Within the central necrosis, a small hole showed the presence of exogeneous material. There were no findings consistent with malignancy. The exogenous material displayed a lumen structure which was suspected to be due to larva migrans. A detailed microscopic examination revealed that the larvae had Y-shaped lateral cords, which are specific to anisakid larvae (Fig. 4). However, it was difficult to make a definitive pathological diagnosis, because the larva body had collapsed. Therefore, the slide was sent to the National Institute of Infectious Diseases (NIID) to identify the type of larvae, and genetic examination using PCR method was performed. DNA was extracted from the deparaffinized slide, and the entire internal transcribed spacer (ITS) region (ITS1-28S region) was amplified by PCR using the primers NC5 and NC2 in the first round. Then nested PCR was performed in the second round to amplify the ITS1-28S region using the following primers that were originally constructed: AniT1F1: 5′-GTTGAACAACGGTGA CCAATTTGGC-3′, and AniT1R1: 5′-GAGTGATCCACCGCCAAGATTTGTAC-3′. An amplification product of 174 bp was obtained, and its sequence alignment was consistent with that of *Pseudoterranova decipiens* sensu *lato* but not with *Anisakis simplex* sensu stricto (Fig. 5). Finally the liver lesion was pathologically and genetically diagnosed as hepatic anisakiasis due to *Pseudoterranova decipiens* sensu *lato*. These findings showed the liver lesion was not due to recurrence of testicular cancer. The patient was without recurrence for 2 years and 4 months.

## Discussion and conclusions

This present report is the first to discuss the case of a patient with hepatic anisakiasis caused by *Pseudoterranova decipiens.* It was difficult to differentiate between a metastatic liver cancer and parasitic granuloma simply by using imaging modalities. Laparoscopic partial hepatectomy for pathological examination and genetic identification of anisakiasis by PCR method were useful for making a final diagnosis even when the larvae body in the resected specimen collapses with time.

Most of extra-gastrointetinal anisakiasis are asymptomatic and detected incidentally [11]. Incidental detection of this lesion is difficult to diagnose and differentiate from recurrence in patients with a prior history of malignancy as in the present case.

Anisakiasis can be categorized into four clinical phenotypes, including gastric, intestinal, extra-gastrointestinal, and allergic [1]. Extra-gastrointestinal anisakiasis accounts for 0.45% of all cases of anisakis in Japan [12], and hepatic anisakiasis is particularly rare. Only 8 cases including our current case study have been reported in full text articles as case report with detailed medical history, with an English title, based on results from a keyword search of “hepatic anisakiasis” on the PubMed and Japan Medical Abstracts Society websites (Table 1) [11, 13–19]. All cases were reported from Japan. The median age was 59 (range: 28–70), and the subjects, 6 males and 2 females. In most cases, the tumor was located in the surface of liver and presented a low density mass using the contrast enhanced CT scan. The median size of the tumor was 15 (4–20) mm. In general, clinical diagnosis of the hepatic anisakiasis proves to be difficult. All patients underwent hepatectomy with preoperative diagnosis of the liver tumor such as metastatic liver cancer in 5, intrahepatic cholangiocarcinoma in one, and unclassified liver tumor in one. Among these patients, 4 patients obtained a diagnosis of hepatic anisakiasis by histopathological examination and 4 others including our current case study were diagnosed with genetic identification by using of PCR method. In our current case study, a tumor biopsy was considered to be an option for diagnosis that could make surgery unnecessary. However, the specimen obtained with the biopsy was considered unsuitable for definitive diagnosis because of the possibility that the amount of the larvae sample would not be sufficient to perform both a pathological examination and genetic identification. Because our current case study had a prior history of malignancy, we could not exclude the possibility of metastatic liver cancer. It was reported that a granuloma of anisakiasis showed rim enhancement on CT with infusion hepatic arteriography (CTIHA) which appeared similar to the peritumoral enhancement of metastatic cancer, suggesting potential difficulty in making the differential diagnosis between hepatic anisakiasis and metastatic liver cancer on imaging studies [20]. Laparoscopic partial hepatectomy is a minimally invasive surgery, and in terms of the tumor location, the surface area, as in our current case study, is suitable for this procedure. Therefore, laparoscopic partial hepatectomy is an acceptable option for the definitive pathological diagnosis of hepatic anisakiasis and differentiation from metastatic liver cancer. As for the pathological diagnosis of hepatic anisakiasis, the anisakis larvae generally collapses and forms granuloma after migrating into the liver and this may cause difficulty with diagnosis even after resection, as in our case study. The PCR methods described here are reliable for obtaining a definitive diagnosis of hepatic anisakiasis in the case studies covered in this paper. In terms of sibling species, which caused hepatic anisakiasis, the *Anisakis* sensu *lat*o in three cases, including *Anisakis pegreffii* in two case and *Anisakis simplex sense stricto in one,* were genetically identified, and the present case is first case in which *Pseudoterranova decipiens* sensu *lato* was genetically identified by the PCR method*.*

**Table 1:** Cases of hepatic anisakiasis presenting as a liver tumor including the present case

Case	Author	Age	Sex	Location (surface or not)	Size (mm)	Contrast-enhanced CT	Preoperative diagnosis	Definitive diagnosis	Species
1	Kagei	51	M	unknown	15	None	Metastatic liver tumor	Operation (histopathology)	Not assessed
2	Kawakami	58	M	S8 (surface)	20	Low density (delayed enhancement)	Metastatic liver tumor	Operation (histopathology)	Not assessed
3	Morita	59	F	S2 (surface)	20	Low density	Liver tumor	Operation (histopathology)	Not assessed
4	Ishida	64	M	S8 (surface)	20	Low density (peripheral enhancement)	Intrahepatic cholangiocarcinoma	Operation (histopathology)	Not assessed
5	Hayashi	70	M	S8 (surface)	8	Low density	Metastatic liver tumor	Operation (PCR)	*Anisakis pegreffii*
6	Nogami	44	F	S4 (surface)	15	Low density	Metastatic liver tumor	Operation (PCR)	*Anisakis* simplex sensu stricto
7	Sekoguchi	63	M	S6 (surface)	4	None	None	Operation (PCR)	*Anisakis pegreffii*
8	Our case	28	M	S7 (surface)	12	Low density	Metastatic liver tumor	Operation (PCR)	*Pseudoterranova decipiens* sensu *lato*

Anisakid larvae is commonly classified into two types that have been implicated in human disease: *Anisakis* (sensu *lato*) and *Pseudoterranova* (sensu *lato*). No studies have been reported about the frequency of species in extragastrointestinal anisakiasis. However, Umehara A. et al. reported that 99% of all human anisakiasis are due to *Anisakis simplex* sensu stricto [21].

In general, the invasive capacity of *Pseudoterranova* spp. larvae is very low relative to that of *Anisakis* spp. *Anisakis* spp. are most often implicated in the invasive type of anisakiasis [7, 8, 22]. In contrast, larvae belonging to the *Pseudoterranova* spp. are generally noninvasive, and hepatic anisakiasis due to *Pseudoterranova* spp. is particulary rare. In Japan, a case report described ectopic anisakiasis rapidly forming a mass in the left inguinal area in a 5-year old patient caused by *Psuedoterranova azarasi*. [17]. No reports to date have documented cases of hepatic anisakiasis caused by *Pseudoterranova* spp. To the best of our knowledge, our current case study is the first to show hepatic anisakiasis caused by *Pseudoterranova decipiens*, only diagnosed through the process of genetic identification.

The *Pseudoterranova decipiens* sensu *lato,* referred to as *Pseudoterranova spp.,* species complex consists of at least 5 sibling species which are genetically but not morphologically distinguishable: *P. decipiens* sensu stricto, *P. azarasi, P. cattani, P. krabbei*, and *P. bulbosa* [6]. Among these siblings, *P. decipiens* sensu stricto*, P. azarashi, and P. cattani* were reported to be the sibling species related to human infection [6, 17, 23, 24]. Geographic distribution of the 5 sibling species of the *P. decipiens* complex differs somewhat. *P. azarasi* and *P. bulbosa* are found in the Northwestern Pacific including Japan, *P. decipiens* sensu stricto and *P. krabbei* in the Northeastern Atlantic, *P. decipiens* sensu stricto in the Northwestern Atlantic, and *P. cattani* in the Southeastern Pacific waters [25]. The infective larvae of *P. azarasi* were identified from the tissues of various marine fish, including cod, pollack, and smelt and their adult worms live in the intestines of seals and sea lions in the sea Japan [6]. Based on case reports of human infection and geographic distribution, the clinical isolation of our current case study most likely belongs to the *P. azarasi*, although we could not genetically identify which sibling species it belong to.

In conclusion, we experienced a case of hepatic anisakiasis caused by *Pseudoterranova decipiens* sensu *lato.* Our results indicate that it is difficult to distinguish hepatic anisakiasis from a metastatic liver cancer on imaging studies for the patients with prior history of malignancy. Genetic identification with the PCR method is reliable for obtaining a final diagnosis even when the larvae body in the resected specimen collapses with time.

## Abbreviations

ADC: apparent diffusion coefficient
CT: computed tomography
PCR: polymerase chain reaction
PET: positron emission tomography
